# A novel planar image-based method for bone marrow dosimetry in ^177^Lu-DOTATATE treatment correlates with haematological toxicity

**DOI:** 10.1186/s40658-016-0157-0

**Published:** 2016-09-22

**Authors:** Johanna Svensson, Tobias Rydén, Linn Hagmarker, Jens Hemmingsson, Bo Wängberg, Peter Bernhardt

**Affiliations:** 1 Department of Oncology, Sahlgrenska University Hospital, 41345 Gothenburg, Sweden; 2 Department of Radiation Physics, Sahlgrenska University Hospital, Gothenburg, Sweden; 3 Department of Surgery, Institute of Clinical Sciences, The Sahlgrenska Academy, Sahlgrenska University Hospital, Gothenburg, Sweden; 4 Department of Medical Physics and Biomedical Engineering, Sahlgrenska University Hospital, Gothenburg, Sweden

**Keywords:** PRRT, ^177^Lu-DOTATATE, Dosimetry, Haematological toxicity

## Abstract

**Background:**

^﻿177^Lu-DOTATATE is a valuable treatment option for patients with advanced neuroendocrine tumours overexpressing somatostatin receptors. Though well tolerated in general, bone marrow toxicity can, besides renal exposure, become dose limiting and affect the ability to sustain future therapies. The aim of this study was to develop a novel planar image-based method for bone marrow dosimetry and evaluate its correlation with haematological toxicity during ^177^Lu-DOTATATE treatment. In this study, 46 patients with advanced neuroendocrine tumours were treated with 7.2 GBq (3.5–8.3 GBq) of ^177^Lu-DOTATATE on two to five occasions. Planar gamma camera images were acquired at 2, 24, 48 and 168 h post-injection. Whole-body regions of interest were created in the images, and a threshold-based segmentation algorithm was applied to separate the uptake of ^177^Lu-DOTATATE into high and low uptake compartments. The conjugate view method was used to quantify the activity, the accumulated activity was calculated and the absorbed dose to the bone marrow was estimated according to the MIRD scheme. Patients were monitored for haematological toxicity based on haemoglobin (Hb), white blood cell (WBC) and platelet (PLT) counts every other week during the treatment period.

**Results:**

The mean absorbed dose to the bone marrow was estimated to 0.20 Gy (0.11–0.37 Gy) per 7.4 GBq of ^177^Lu-DOTATATE, and the mean dose per fraction correlated with a decrease in Hb (*p* = 0.01), WBC (*p* < 0.01) and PLT (*p* < 0.01) counts. The total mean absorbed dose to the bone marrow was 0.64 Gy (0.30–1.5 Gy) per 24 GBq (8.2–37 GBq) of ^177^Lu-DOTATATE and also correlated with a decrease in Hb (*p* < 0.01), WBC (*p* = 0.01) and PLT (*p* < 0.01) counts.

**Conclusions:**

The planar image-based method developed in this study resulted in similar absorbed doses to the bone marrow as reported in earlier studies with blood-based bone marrow dosimetry. The results correlated with haematological toxicity, making it a promising method for estimating bone marrow doses in ^177^Lu-DOTATATE treatment without the need for blood and urine sampling.

## Background

Treatment with ^177^Lu-labelled somatostatin analogues (^177^Lu-DOTATATE) for patients with advanced neuroendocrine tumours has been proven safe and efficient [1–3]. The bone marrow is considered the most important dose-limiting organ besides the kidneys [1, 2, 4]. Clinical protocols for ^177^Lu-DOTATATE treatment routinely include mean absorbed dose estimates for the kidneys [1, 2, 5], which usually decide the number of fractions given because of a defined dose limit, even though clinically relevant renal toxicity seldom is observed [6, 7]. In contrast, bone marrow dosimetry is only performed occasionally, and instead haematological toxicity monitored in terms of haemoglobin (Hb), white blood cell (WBC) and platelet (PLT) counts serves as a surrogate of bone marrow function. Haematological toxicity is often present during ^177^Lu-DOTATATE treatment and is accepted, as it usually is mild and transient [1, 2, 8]. However, serious haematological toxicity (grade 3–4 according to NCI CTCAE version 3.0) is reported in 10 % of treated patients, and long-term effects in terms of myelodysplastic syndrome or leukaemia are described in 1–2 % of treated patients [1, 9].

The most accepted mean absorbed dose limit to the bone marrow for PRRT is 2 Gy [1, 10], which was originally set based on experiences with ^131^I therapy for the treatment of thyroid cancer [11–13]. The methods used to estimate the absorbed dose to the bone marrow in radionuclide therapy is based on the MIRD scheme [14], which includes the absorbed dose from charged particles and photons in the bone marrow itself (self-dose) and the absorbed dose due to the photon radiation from activity distributed in the remainder of the body (cross-dose). Most often described is an indirect method to estimate the absorbed dose that includes repeated blood sampling after therapy to obtain activity concentration estimates, the acquisition of post-therapeutic gamma camera images and sometimes urine sampling (Table 1) [2, 15–18]. This indirect method for bone marrow dosimetry was first described and utilised for radioimmunotherapy [19] and later for PRRT [16, 17].

**Table 1:** Studies on blood and image-based bone marrow dosimetry in ^177^Lu-DOTATATE treatment

Author, year	Number of patients	Method	BM dose (Gy/7.4 GBq)
Wehrmann, 2007	27	Blood, imaging	0.30
Forrer, 2009	13	Blood, urine, imaging	0.25
Bodei, 2011	12	Blood, imaging	0.25
Sandström, 2013	200	Blood, urine, imaging	0.12 median
Jackson, 2013	28	Imaging	0.11–0.26
Bergsma, 2016	23	Blood, urine, imaging	0.50
Present study, 2016	46	Imaging	0.20

*No* number *BM* bone marrow

Blood-based dosimetry relies on the assumption of a specific ratio between the activity concentrations in blood and bone marrow. This ratio has been considered to be close to 1 for radionuclide therapy using peptides, assuming low or no specific binding of the radionuclide in the bone marrow [20], which was confirmed in a study with ^177^Lu-DOTATATE in which bone marrow and blood samples collected at the same time points were analysed [16].

Changes in Hb, WBC and PLT counts after treatment do not necessarily describe the magnitude of the radiation exposure, as the estimated dose to the bone marrow not has been proven to correlate with haematological toxicity [9, 16]. Reasons for this are both uncertainties in the methods used to estimate the dose, and that several other factors affect the haematological response, including baseline WBC counts, patient age, renal function, previous chemotherapy, large tumour burden, bone metastases and splenectomy [8, 9, 18, 21]. Regardless of the dose-response relationship, an estimation of the mean absorbed dose to the bone marrow in PRRT must be considered important because its viability will set the limits for future therapies affecting the bone marrow and affect the risk of developing serious long-term toxicity.

The aim of our study was to develop a novel method for bone marrow dosimetry based on planar post-therapy imaging, without the need for blood and urine sampling, and to investigate a possible correlation with haematological toxicity.

## Methods

### Patients and treatment characteristics

A total of 46 patients at Sahlgrenska University Hospital, who were diagnosed with advanced neuroendocrine tumours, were included in this retrospective study. The study was approved by the Regional Ethics Review Board in Gothenburg and performed in accordance with the principles of the Declaration of Helsinki and national regulations. The need for written informed consent was waived. Patients considered for treatment had a progressive disease; clinically or radiologically. They were judged to have tumours over-expressing somatostatin receptors (i.e. uptake exceeding physiological liver uptake) based on somatostatin receptor scintigraphy (Octreoscan®). ^177^Lu-DOTATATE was administered at an activity of 7.2 ± 1.3 GBq (3.5–8.3 GBq) on two to five occasions, approximately 8 weeks apart, to a total mean activity amount of 24 ± 7.2 GBq (8.2–37 GBq). Each fraction was given as a 30-min intravenous infusion coadministered with kidney-protective amino acids (2.5 % lysine and 2.5 % arginine in 1 L of 0.9 % NaCl; infusion rate 250 mL/h). The number of fractions was decided from received renal dose (limit 27 Gy) or persisting haematological toxicity or disease progression.

### Image acquisition

For this planar dosimetry method based on the conjugate view method, anterior and posterior planar images were acquired at 2, 24, 48 and 168 h post-injection (p.i.). A SPECT/CT was acquired 24 h p.i. for calibration purposes. The gamma cameras were a Picker IRIX (Marconi, Phillips, Holland) and a Millennium VG Hawkeye (General Electric Medical Systems, Milwaukee, WI, USA) equipped with medium energy parallel-hole collimators. Planar whole-body scans were performed with a scanning time of 10 min and a 20 % energy window over the 208-keV photon peak. Measurement over the 113-keV photon peak was omitted due to the high scattering fraction in the data. No further scatter correction was included in the protocol. The SPECT image acquisition used the same energy setting and was performed with 30-s frame duration for 120 projections. The CT images were acquired using 140-kV tube voltage, 2.5 mAs, and a rotation speed of 2.6 rpm. SPECT reconstructions were performed using the ordered subset expectation maximum (OSEM) algorithm with two iterations, 10 subsets and post-filtering. No scattering window was used. The effective attenuation coefficient and the sensitivity for the gamma cameras were determined by scintigraphy of a planar ^177^Lu source placed at different depths in a phantom of tissue equivalent material. A region of interest was drawn around the source in the planar images and the counts recorded after which a monoexponential curve was fitted to the number of counts versus depth data.

### The planar two-compartment method

The method for planar image dosimetry was based on a two-compartment approach that separated the high uptake area from the low uptake area in geometric mean images produced from the anterior and posterior whole-body images. To avoid operator dependence, an automated algorithm was created for the compartment separation. In this automated algorithm, a Butterwort low pass filter at order 2 with a cut-off frequency of 0.03 filtered the geometric mean images, and a contour line algorithm was used to obtain the whole-body region of interest (ROI). Filtering the images was especially important for the 7 day p.i. images, as the noise level would otherwise hamper the accuracy of the whole-body outline. The whole-body ROI was then transposed onto the unfiltered geometric mean images. In the whole-body ROI, the pixels with a value (counts) higher or equal to a specific value (threshold) were identified with a threshold algorithm. This was done for all pixel values, from the maximum value and down to 0. At each threshold value, the number of connected pixel regions (i.e. uptake foci) was determined in the whole-body by a connected-component labelling algorithm [22]. The number of uptake foci (NUF) was calculated at each threshold value, generating a distribution of NUF versus a threshold index (ThI) defined as

1 $$ \mathrm{T}\mathrm{h}\mathrm{I}=\frac{C_{\max}\kern0.35em  - {C}_{\mathrm{thr}}}{C_{\max }} $$

where *C*_max_ is the maximum pixel value in the whole-body ROI and *C*_thr_ is the pixel threshold value. The NUF was normalized (nNUF) to the maximum number of uptake foci and could then be described as a function of ThI, ranging from 0 to 1. The algorithm was implemented into the medical physics, oncology and nuclear medicine image research platform at the Sahlgrenska Academy (PhONSAi) [22].

The distribution of nNUF versus ThI was analysed for all images to define an appropriate nNUF threshold value separating the whole-body into high and low uptake compartments. The high uptake compartment includes organs with physiologically or pathologically high somatostatin receptor expression, such as the liver, spleen, kidneys and tumours. The low uptake compartment corresponds to the remainder of the body. The defined nNUF threshold value was applied to all time points and all patients.

### Dosimetry

The activity in the two compartments was quantified according to the conjugate view method and time-activity curves created [23]. For the conjugate view calculations, the patient-specific thickness of the high uptake compartment was determined over the abdomen by a high-resolution CT and the activity assumed to be concentrated to a volume with a general organ thickness determined from a series of measurements in SPECT images (8 cm). The weight of the high uptake compartment was determined by assuming a density of unity and calculating the volume from the abdominal thickness and the area of the high uptake compartment. In the low uptake compartment, the activity was assumed to be uniformly distributed, and the thickness was determined as the patient-specific weight minus the calculated weight of the high uptake compartment divided by the area of the low uptake compartment. The accumulated activity in the under- and overlying tissue of the high uptake compartment was added to the low uptake compartment.

When four data points were available, the data for the low uptake compartment was fitted to a bi-exponential function, and when 3 data points were available to a mono-exponential function. For the high uptake compartment, data from the 2 and 24 h p.i. images were fitted to a linear function and data from 24 h p.i. and later time points were fitted to a mono-exponential function. The curves were extrapolated to the time of injection (*t* = 0). The accumulated activity was determined by calculating the area under the curve.

SPECT/CT measurements were performed to verify the relationship between the activity concentration in the bone marrow and the low uptake compartment, which was considered to consist mainly of muscle, fat and bone. This was achieved by applying one volume of interest (VOI) in the centre of a lumbar vertebra (L4) to represent the bone marrow and another VOI symmetrically located around L4 to represent the low uptake compartment.

The partial volume effect was determined, and corrected for, by an experimentally determined recovery correction (RC) factor. For this, the bone marrow VOI described above, was placed in the CT image. Activity was then placed in the VOI, and the photon emission and the photon absorption within and outside the VOI was simulated by the Monte Carlo program implemented in the image platform PhONSAi [22].

The ratio between the recovery corrected activity concentration in the bone marrow VOI and the activity concentration in the VOI representing the low uptake compartment was determined from 53 SPECT/CT images of 15 patients without known tumour infiltration in the bone marrow or the tissue surrounding the VOI. To have similar inscattering conditions, the tissue directly surrounding the bone marrow VOI was chosen, representing the low uptake compartment. The tissue of these VOIs was dominated by muscle, fat and connective tissue, with some bone also included, which may be considered representative of the low uptake compartment of the whole body. The ratio of the activity concentrations in the bone marrow and the low uptake compartment was assumed to be constant over time. L4 was chosen because it could be defined on CT to its size and horizontal orientation.

The mean absorbed dose to the bone marrow (*D*_bm_) was calculated as the sum of the self-dose from the charged particles in the bone marrow itself and the cross-doses (γ radiation) from the high and low uptake compartments as follows:

2 $$ {D}_{\mathrm{bm}}={\overset{\sim }{A}}_{\mathrm{bm}}\times {S}_{\mathrm{bm}\leftarrow \mathrm{b}\mathrm{m}}+{\overset{\sim }{A}}_{\mathrm{high}}\times {S}_{\mathrm{bm}\leftarrow \mathrm{high}}+{\overset{\sim }{A}}_{\mathrm{low}}\times {S}_{\mathrm{bm}\leftarrow \mathrm{low}} $$

where the accumulated activity in the bone marrow (*Ã*_bm_) was derived from the accumulated activity in the low uptake compartment (*Ã*_low_) and adjusted by the calculated ratio of the activity concentrations in the bone marrow and the low uptake compartment. The *S* values were obtained from the specific absorbed fractions defined on the RADAR website [24]. The *S* value for the gamma contribution from the high uptake compartment was calculated as a weight-weighted mean of the *S* values for the liver, spleen and kidneys (*S*_bm←high_), and from the low uptake area as a mean of the *S* values for muscle and bone (*S*_bm←low_).

### Monitoring bone marrow function

Bone marrow function was monitored during treatment in terms of Hb, WBC and PLT counts in blood every other week. To evaluate whether the mean absorbed dose to the bone marrow affected the development of haematological toxicity, the mean absorbed dose per fraction and the total absorbed dose to the bone marrow were compared to the nadir values for Hb, WBC and PLT counts during treatment.

### Statistical analysis

The association of a decrease in blood counts from baseline values in the study population and the absorbed dose to the bone marrow was examined by analysis of variance (ANOVA) in linear regression analysis. All imaging data were fitted using MATLAB® and the coefficient of determination (*R*^2^) calculated. *p* values <0.05 were considered significant.

## Results

**Fig. 1:**
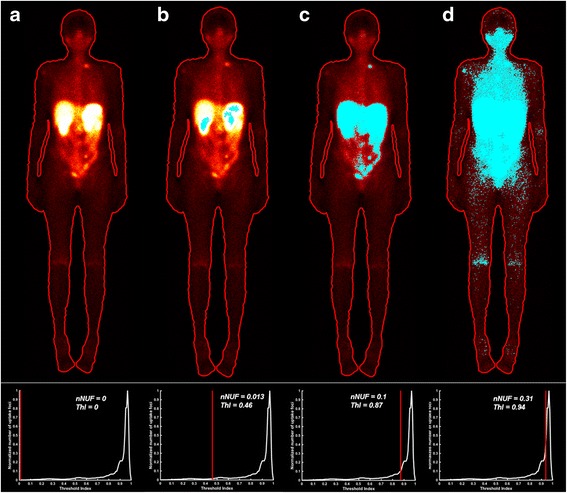
Illustration of the segmentation technique used to divide the activity uptake into a high and a low uptake compartment. A region of interest of the whole body (**a**). The automated segmentation tool includes foci in the whole body starting with the highest uptake foci to the left on the *x*-axis (**b**). Moving to the right, more and more foci will be included (normalized number of uptake foci on the *y*-axis) until a threshold is reached (**c**), just before the number of foci increases and the noise is included (**d**). The cut-off between the high and low uptake compartments (nNUF = 0.1) is set at the threshold value illustrated in (**c**) *nNUF* normalized number of uptake foci *ThI* threshold index

The methodology for the planar image dosimetry was straightforward and robust. Visual analysis of nNUF versus ThI dependence revealed a similar pattern in all images (Fig. 1). The number of foci gradually increased as the ThI increased, until a threshold was reached and the number of foci increased substantially. This threshold value, used as the cut-off between the high and low uptake compartments, was 0.1, implying that 10 % of the foci are included in the high uptake compartment and the remaining 90 % in the low uptake compartment. The sensitivity of the threshold value was tested for by changing its value between 0.05 and 0.25 and the mean absorbed bone marrow doses varied up to 10 % in this interval. No significant difference was observed in the coefficient of variation (8.5–8.9 %) for the individual mean absorbed doses when varying the threshold value between 0.05 and 0.25.

**Fig. 2:**
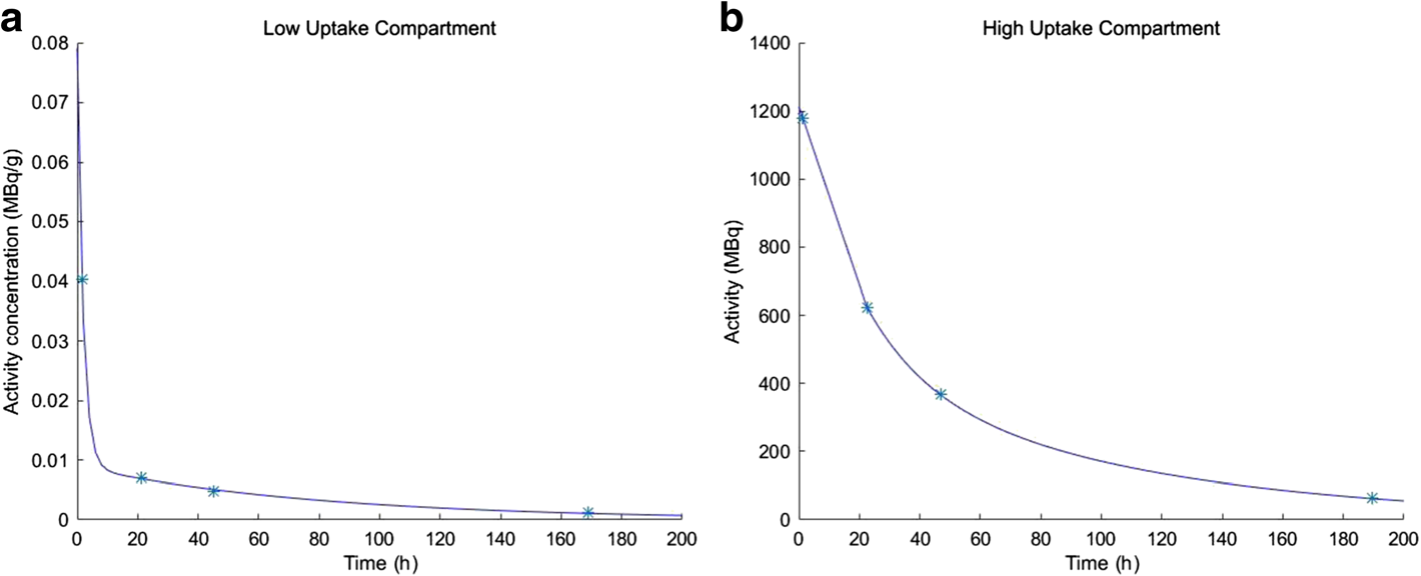
An example of the clearance of the low uptake compartment illustrated as a time-activity concentration curve (*t*_½ 1stphase_ 2.4 h, *t*_½ 2ndphase_ 61 h for all patients) (**a**), and the clearance of the high uptake compartment illustrated as a time-activity curve (*t*_½ 2ndphase_ 69 h for all patients) (**b**)

Time-activity curves for the low uptake compartment were fitted to a bi-exponential function when four time points were available (165/174 fractions; Fig. 2). For patients with only three data points, the time-activity curve was fitted to a mono-exponential function. The effective half-lives for the first and second phases in the low uptake compartment were 2.4 ± 1.7 h (1.0–11 h) and 61 ± 9.2 h (43–89 h), respectively. For the high uptake compartment, the time-activity curves for all patients were fitted to a linear function for the first phase and a mono-exponential function for the second phase, with an effective half-life for the second phase of 69 ± 14 h (12–102 h) (Fig. 2). In 28 % of these time-activity curves, there was an initial uptake phase (0–24 h). Most of the accumulated activity in the whole body, 63 ± 11 % (23–90 %), was deposited in the high uptake compartment and 37 ± 11 % (10–77 %) in the low uptake compartment.

**Fig. 3:**
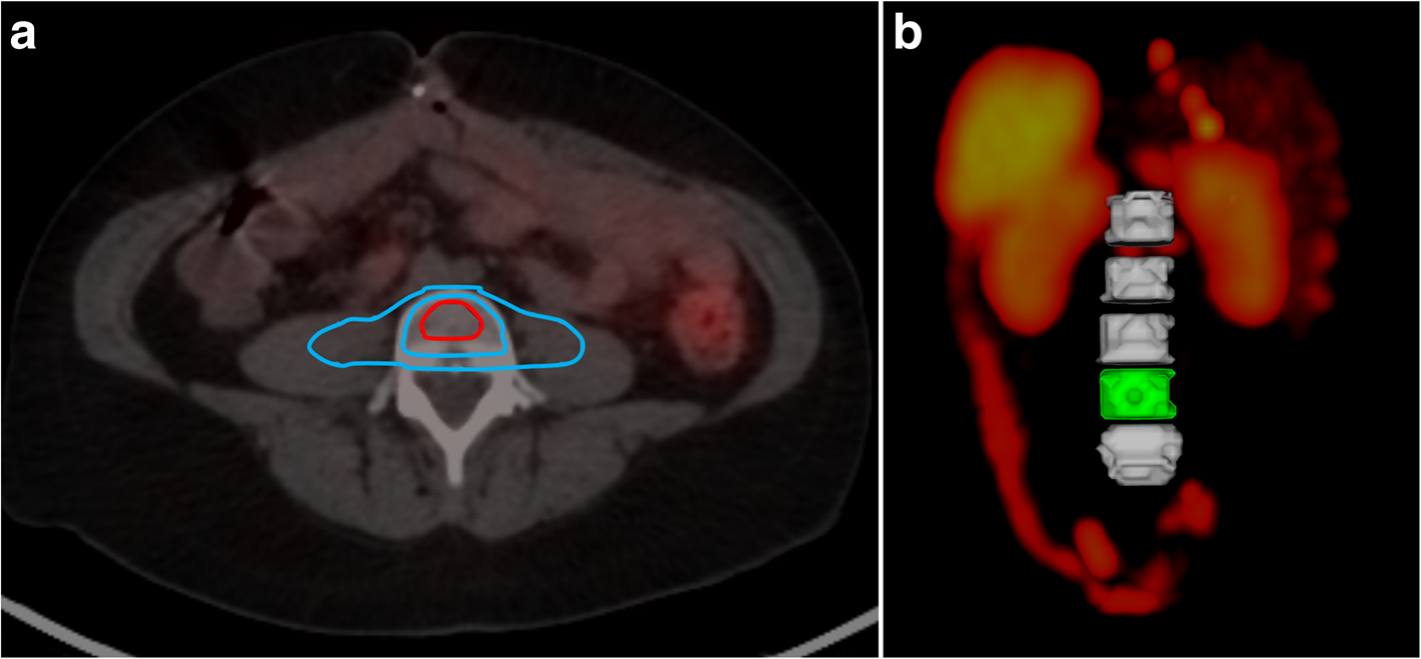
Cross-section of an abdominal CT scan with a delineated volume of interest (VOI) around the bone marrow in the lumbar spine (L4) and a surrounding VOI representing the low uptake compartment in the whole body (**a**) and a posterior view of a SPECT image illustrating the localisation of lumbar spines L1–L5 and their relation to abdominal organs (**b**)

Figure 3 illustrates a delineated bone marrow VOI (*V*_mean_ = 8 mm^3^) in the lumbar spine and a surrounding VOI (*V*_mean_ = 34 mm^3^) representing the low uptake compartment in a horizontal CT slice. The recovery corrected ratio between the bone marrow and low uptake compartment activity concentrations was estimated to 1.8 (1.4–2.8).

**Fig. 4:**
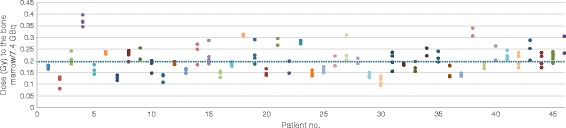
The variation in absorbed dose to the bone marrow between treatments for the 46 individual patients (cv = 0.08). The *gray-dotted line* represents the mean absorbed dose per treatment for all patients, 0.20 Gy. *No* number

The mean absorbed dose to the bone marrow for the 46 patients included in this study was estimated to 0.20 ± 0.05 Gy (0.11–0.37 Gy) per 7.4 GBq of ^177^Lu-DOTATATE. The variation in mean absorbed dose between treatments is illustrated in Fig. 4. The coefficient of variation (CV) for the individual mean absorbed bone marrow doses was 0.08. For all fractions, the mean total absorbed dose was 0.64 ± 0.22 Gy (0.30–1.5 Gy) from a total amount of 24 ± 7.2 GBq (8.2–37 GBq) ^177^Lu-DOTATATE. The self-dose dominated, contributing 85 ± 4.0 % (61–92 %) of the total absorbed dose to the bone marrow. The cross-doses from the high and low uptake compartments contributed 8.9 ± 3.6 % (2–23 %) and 5.8 ± 1.9 % (1.4–25 %) of the total dose, respectively.

**Fig. 5:**
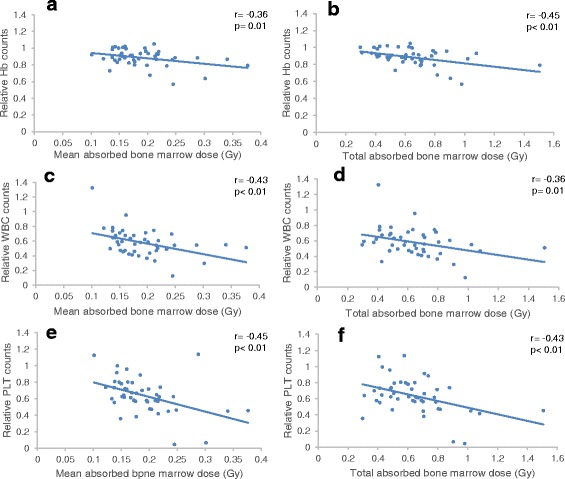
Mean absorbed doses and total absorbed doses to the bone marrow correlated with the decrease in Hb (**a**, **b**), WBC (**c**, **d**) and platelet (**e**, **f**) counts respectively during ^177^Lu-DOTATATE treatment

Correlations were found between the calculated mean absorbed dose to the bone marrow per treatment and the decrease in Hb (Pearson *r* = −0.36, *p* = 0.01), WBC (*r* = −0.43, *p* < 0.01) and PLT counts (*r* = −0.45, *p* < 0.01). In addition, a higher total absorbed dose to the bone marrow was associated with the decrease in Hb (*r* = −0.45, *p* < 0.01), WBC (*r* = −0.36, *p* = 0.01) and PLT counts (*r* = −0.43, *p* < 0.01; Fig. 5).

## Discussion

This novel planar image-based method for bone marrow dosimetry yields estimated mean absorbed doses of 0.20 Gy per 7.4 GBq of ^177^Lu-DOTATATE, which is comparable to other published series on blood-based bone marrow dosimetry for this treatment (Table 1). The correlation with haematological toxicity is significant but not patient-specific due to large variations in the established correlation. There are several possible reasons for the lack of a more apparent dose-response relationship with this method and also with blood-based dosimetry. Blood-based methods have uncertainties, including the timing and handling of the blood samples and calibration of the instruments. The method developed in this study to divide the whole-body uptake into two compartments in an automated manner seems feasible but will share the uncertainties included in all planar dosimetry approaches. Furthermore, both this image-based method and blood-based dosimetry involve an indirect estimation of the activity concentration in the bone marrow and choosing a ratio for the compartment that is quantified to the bone marrow. The ratio in this study was based on a mean value for the ratio between the measured activity concentration in a lumbar vertebra (L4), representing the bone marrow, and a VOI drawn around the same vertebra, representing the low uptake compartment in the 24-h SPECT in patients without bone metastases. The ratio is then applied to all patients, possibly underestimating the ratio for patients with extensive bone involvement. Any inscattering is assumed to be equal to the two VOIs; however, this will lower the ratio between the two VOIs, which may further underestimate the obtained ratio. Another aspect is the possible difference in the kinetics of the radiopharmaceutical in the low uptake compartment compared to the bone marrow, which may lead to an overestimation or underestimation of the absorbed dose to the bone marrow using a fixed ratio.

A direct measurement of the activity concentration in the bone marrow is described for ^177^Lu-DOTATATATE, and a ratio of 1 was found to the activity concentration in blood at the same time point [16]. Following the activity concentration and its kinetics directly by frequent bone marrow sampling could be considered the most accurate method but is not practicable. Even with only one bone marrow sample, this method would not describe the possible regional differences in bone marrow distribution or activity concentration [25].

The self-dose dominated the contributions to the absorbed dose to the bone marrow, just as Sandström et al. observed, though to a lesser extent (85 versus 65 %) [17], using blood-based dosimetry. In contrast, Forrer et al. calculated that approximately one third of the absorbed dose to the bone marrow was derived from the self-dose in blood-based dosimetry [16]. In that study, the contribution from the remainder of the body was intentionally overestimated from the activity quantification in urine sampled for the first 24 h p.i.

Another aspect to consider when comparing dose estimates between studies is that different *S* values may be used, from either the original MIRD Pamphlet No. 11 or newer phantoms, including data published on the RADAR website, where *S* values have been reduced in general [24]. The weighted *S* values produced in this study to calculate the cross-doses from the high and low uptake compartments are simplified but should be useful in most cases, as their contribution was generally low.

Further optimization of the presented method would be valuable to explore whether improved correlations can be found for the absorbed dose to the bone marrow and haematological toxicity. Strategies should include the use of simulated planar images from different sized phantom measurements to explore the optimal threshold NUF and selecting more patient-specific *S* values for cross-doses. The use of different scattering correction techniques and methodologies for attenuation correction, such as scout images, is another factor to explore.

Pure image-based methods to estimate the absorbed dose to the bone marrow in radionuclide therapy have been described previously, and originally for radioimmunotherapy, by Siegel et al. [26] based on quantification of the accumulated activity in the sacral region in the post-therapeutic planar images. Forrer et al. [16] also evaluated an image-based method to estimate the accumulated activity of ^177^Lu-DOTATATE in the bone marrow by quantifying the activity concentration in the thoracic spine from SPECT images after therapy. However, the authors concluded that the spine uptake was too close to background values. In a recent study, an image-based method for bone marrow dosimetry in ^177^Lu-DOTATATE treatment was presented using repeated SPECT images for voxel-based quantification of the radioactivity in the lumbar spine [27], but the method was not verified against haematological toxicity. Despite the uncertainties in the presented method, it showed a dose-response relationship between absorbed bone marrow dose and haematological toxicity according to Hb, WBC and PLT counts for both mean and total absorbed dose. One previous study, recently performed by Bergsma et al., observed a correlation between cumulative bone marrow dose and WBC and PLT counts, not Hb, in a sub-group analysis of 14 patients [18].

The estimation of the bone marrow exposure needs to be as accurate as possible, but the haematological response will only be fully understood when also other clinical data for the patient is considered, as renal function, tumour burden, baseline blood values and age [18, 21] as well as the individual radiosensitivity.

The correlation with haematological toxicity encourage further development of the method described for bone marrow dosimetry to increase the accuracy of absorbed dose estimates and the correlation to haematological response. A sub-study including blood-based dosimetry to compare the method for the individual patient is initiated in the ongoing prospective phase II study on ^177^Lu-DOTATATE treatment for patients with progressive neuroendocrine tumours (ILUMINET, EudraCT 2011-000240-16)

## Conclusions

This study presents a novel image-based method for bone marrow dosimetry depending mainly on planar imaging with correlation to haematologic toxicity. The results encourage to further development of the method, aiming to increase the accuracy in the absorbed dose estimates and its correlation to haematological response. The need for individual BM dosimetry grows as the total amount of ^177^Lu-DOTATATE given tends to increase and future treatment options make it necessary to preserve bone marrow function.
